# The Role of the Microbiome in the Pathogenesis and Treatment of Ulcerative Colitis—A Literature Review

**DOI:** 10.3390/biomedicines11123144

**Published:** 2023-11-25

**Authors:** Gabriela Świrkosz, Aleksandra Szczygieł, Katarzyna Logoń, Martyna Wrześniewska, Krzysztof Gomułka

**Affiliations:** 1 Student Scientific Group of Adult Allergology, Wroclaw Medical University, 50-369 Wrocław, Poland; gabriela.swirkosz@student.umw.edu.pl (G.Ś.); katarzyna.logon@student.umw.edu.pl (K.L.); 2 Clinical Department of Internal Medicine, Pneumology and Allergology, Wroclaw Medical University, 50-369 Wrocław, Poland; krzysztof.gomulka@umed.wroc.pl

**Keywords:** ulcerative colitis, microbiome, gut microbiome, prebiotics, probiotics, treatment

## Abstract

Ulcerative colitis (UC) is a chronic inflammatory bowel disease affecting the colon and rectum. UC’s pathogenesis involves colonic epithelial cell abnormalities and mucosal barrier dysfunction, leading to recurrent mucosal inflammation. The purpose of the article is to show the complex interplay between ulcerative colitis and the microbiome. The literature search was conducted using the PubMed database. After a screening process of studies published before October 2023, a total of 136 articles were selected. It has been discovered that there is a fundamental correlation of a robust intestinal microbiota and the preservation of gastrointestinal health. Dysbiosis poses a grave risk to the host organism. It renders the host susceptible to infections and has been linked to the pathogenesis of chronic diseases, with particular relevance to conditions such as ulcerative colitis. Current therapeutic strategies for UC involve medications such as aminosalicylic acids, glucocorticoids, and immunosuppressive agents, although recent breakthroughs in monoclonal antibody therapies have significantly improved UC treatment. Furthermore, modulating the gut microbiome with specific compounds and probiotics holds potential for inflammation reduction, while fecal microbiota transplantation shows promise for alleviating UC symptoms. This review provides an overview of the gut microbiome’s role in UC pathogenesis and treatment, emphasizing areas for further research.

## 1. Introduction

Ulcerative colitis (UC) is a chronic, idiopathic, non-specific inflammatory disorder affecting the colon and rectum, categorized as an inflammatory bowel disease (IBD) [1]. Its clinical manifestations encompass symptoms such as diarrhea, abdominal discomfort and presence of blood in stool [2]. The pathogenesis of UC is associated with abnormalities in colonic epithelial cells, the mucus barrier, and the epithelial barrier [3,4,5,6] with a recurring pattern of mucosal inflammation spreading from the rectal region to the upper parts of the colon [7,8]. UC does not exhibit a sex-based prevalence. The disease onset typically falls within the age range of 30 to 40 years [9,10]. Most UC patients experience a mild to moderate disease course, characterized by periods of heightened activity upon diagnosis and subsequent intervals of remission [11]. Hospitalization due to UC is common, with nearly half of all patients requiring UC-related hospitalization at some point during their ailment [12]. UC severely affects the quality of life of afflicted individuals and unfortunately it is associated with an elevated risk of colorectal cancer, as well.

Factors found to impact the development of UC include genetic predisposition, gut microbiota dysbiosis, and environmental factors [13,14,15]. Approximately one in ten individuals diagnosed with UC has a relative suffering from IBD [16]. Genetic loci linked to a higher susceptibility to UC include the human leukocyte antigen and genes related to barrier function—HNF4A and CDH1 [17,18]. Notably, cigarette smoking is a strong risk factor for developing UC [19,20,21]. Certain medications, especially hormonal therapy and NSAIDs, increase the risk of UC onset [22,23,24], while breastfeeding reduces it [25]. 

Intestinal microbiota imbalance can result in a decline of the pivotal functions of the gut, subsequently elevating the risk of UC onset, among many other diseases [26,27,28]. Dysbiosis of the gut microbiota is a critical contributor to UC development, typically featuring diminished bacterial diversity in the intestines [29]. Patients suffering from UC exhibit reduced gut microbiota diversity, a decreased prevalence of Firmicutes, and elevated rates of Gamma-proteobacteria and Enterobacteriaceae [30,31,32], which is what fecal microbiota transplantation (FMT) is aiming at [33,34,35]. 

The goal of UC management is to induce and maintain remission with the prevention of colectomy and colorectal cancer in mind [36,37,38]. Treatment choices are based mainly on case severity, while watchfully observing the patient’s response [39,40]. UC can be managed through pharmacological interventions, including aminosalicylates, glucocorticoids, biological agents, and immunosuppressants [4,14,41,42,43,44,45]. Nevertheless, even with this extensive array of therapeutic options, there are instances where patients fail to respond to treatment or experience suboptimal therapeutic effects. Alternative, novel therapies for such cases include dietary changes, adding a probiotic, prebiotic or a synbiotic to current medication regimes, or FMT. 

In this review we aimed to summarize recent findings on the impact of the gut microbiome in ulcerative colitis pathogenesis and treatment, underlining the areas that require further research. Our review presents a comprehensive approach to the microbiota in UC, incorporating insights from the latest scientific literature. Our objective was to address both the pathogenesis and alterations in the microbiome, along with factors influencing its composition, as well as therapeutic aspects.

## 2. Intestinal Microbiota in the Context of Ulcerative Colitis

**Figure 1:**
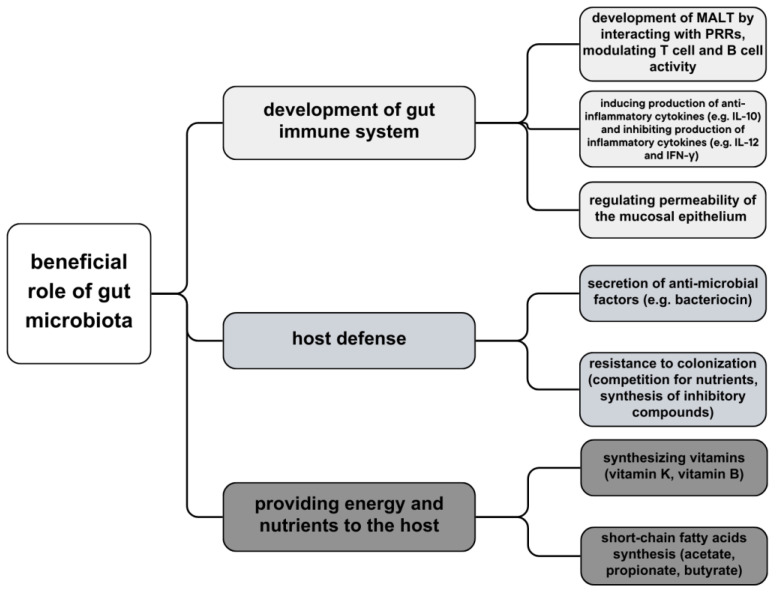
Beneficial role of gut microbiota—development of immune system, host defense and providing energy and nutrients.

Each human body is inhabited by a large amount of commensal microbiota, consisting of bacteria, viruses, and fungi, with its primary location and highest density in the gastrointestinal tract (increasing from duodenum to distal colon) [46]. Recent data reveals a diverse assemblage exceeding 1000 distinct microbial species inhabiting the gastrointestinal tract with its microbial genetic composition more than a hundredfold of the human genome. It is a significant argument for the theory that microbiota warrants consideration as a vital organ within the human body [47]. Intestinal bacteria can be divided into three groups, depending on aerobiosis or anaerobiosis: aerobic, facultatively anaerobic, and anaerobic bacteria—dominant microbiota of the intestine. Anaerobic bacteria, such as *Bifidobacterium, Bacterioides,* and *Peptococcus*, play key roles in nutrition and immune regulation. These bacterial strains act as a barrier to impede the intrusion of pathogens into the lamina propria layer. They elicit a controlled inflammatory response, thereby stimulating the intestinal mucosa and fostering the development and enhancement of the intestinal immune system. Moreover, certain bacterial species exhibit the capacity to modulate physiological metabolic activities; for instance, *Propionibacterium freudenreichii ET-3* demonstrates the capability to synthesize substantial quantities of vitamin K2 precursor which not only holds potential in activating aromatic receptors but also participates in substance metabolism [27]. Certain commensal bacteria exert a direct inhibitory influence on intestinal pathogens through competitive mechanisms for nutrient resources or by inducing the synthesis of inhibitory compounds. *Bacteroides thetaiotaomicron*, an abundant anaerobic resident of the colonic environment, consumes carbohydrates essential for the growth of *Citrobacter rodentium*, thereby facilitating competitive exclusion of the pathogen from the intestinal lumen. Additionally, *B. thuringiensis* secretes bacteriocins with specific antagonistic activity against spore-forming bacteria of the *Bacilli* and *Clostridia* classes, including notable targets such as *Clostridium difficile* [48] (Figure 1).

Facultative anaerobic bacteria and intestinal nondominant bacteria (e.g., *Enterococcus, Enterobacter*) inhabit the host; they are harmless when the microecological balance is maintained and potentially harmful in a disturbed gut environment. Pathogens, such as *Pseudomonas* and *Proteus*, in a balanced microecology, are nonpathogenic, usually inhabit the host organism for short periods, and appear in small populations. However, in the situation of diminution of intestinal-dominant microbiota and microbiome dysbiosis, they might have a detrimental effect on the immune system, expose the host to other conditional pathogens, or allow other diseases to develop [27].

Bacteria occur mostly on the surface of intestinal mucosa, forming a layer of biofilm that produces nutrients, and affects tissue permeability as well as the intestinal immune system [27,49]

A principal role of intestinal microbiota is to synthesize vitamins, metabolize proteins and carbohydrates and contribute to the development of GALT—gut-associated lymphoid tissue—by producing an enormous number of metabolites that regulate interactions between epithelium and immune cells [50,51]. The microbiota residing in the distal gastrointestinal tract demonstrates the capacity for endogenous biosynthesis of vitamin K as well as the majority of water-soluble B vitamins, including biotin, cobalamin, folates, nicotinic acid, pantothenic acid, pyridoxine, riboflavin, and thiamine [52]. The microbial fermentation of dietary non-digestible carbohydrates gives rise to the production of beneficial short-chain fatty acids, notably acetate, propionate, and butyrate, which serve as principal anions in the colon. Butyrate, in particular, functions as a primary energy substrate for colonic epithelial cells and exhibits anticarcinogenic and anti-inflammatory properties [48,53]. In the intestinal mucosal surface, a first-line defense of mucosa-associated lymphoid tissue (MALT), and immune tolerance towards microorganisms is established. Gut microbiota is recognized, which starts with pattern recognition receptor systems (PRRs): nucleotide-binding oligomerization domain receptors (NODs) and toll-like receptors (TLRs). These receptors appear on the surface of intestinal epithelial cells, macrophages, and intestinal dendritic cells and distinguish microbe- or pathogen-associated molecular patterns (MAMPs or PAMPs) on pathogens and commensals; if the microbe passaged through the epithelium, immunologic response would be targeted against it [51]. Host microbial bacteria decrease the migration of phagocytes transferring bacterial antigens to local lymphoid tissues, resulting in a lack of activation of T-cells and B-cells. Commensal microbes also accelerate goblet cell differentiation and epithelial mucosa manufacture [54].

Gut microbiota also regulates permeability within the distinct lamina of intestinal mucus. The mucosal epithelium of the intestine comprises enterocytes (absorptive cells), goblet cells, and Paneth cells. Goblet cells secrete hyperglycosylated mucin MUC2 [55]. Mucin provides static shielding protection as well as immunogenicity of intestinal antigens by imprinting DCs towards anti-inflammatory reactions in the intestine. Gut microbial signals (e.g., metabolite indole) promote the strengthening of the epithelial barrier by increasing the amount of tight junctions and cytoskeletal proteins in the epithelium [56].

**Figure 2:**
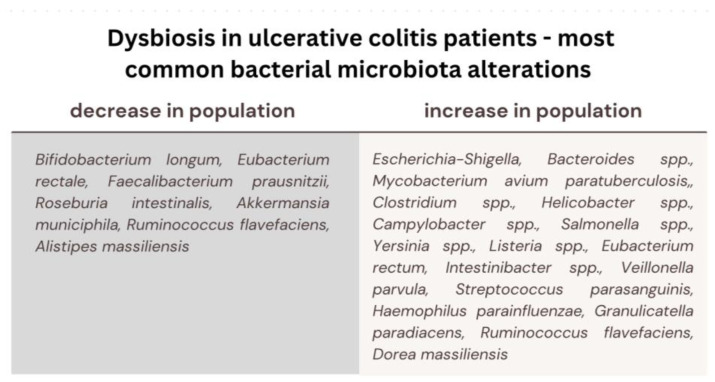
Most common bacterial microbiota alterations in ulcerative colitis patients; decrease in the population of the beneficial microbiota (participating in the development of immune system, host defense and providing energy and nutrients) and an increase in the population of the detrimental microbiota (causing damage to intestinal cells, promoting inflammatory cytokines, initiating immune response).

In the context of enteric colonization by pathogenic microorganisms, there is a potential outcome characterized by heightened intestinal permeability, facilitating the translocation of bacterial antigens from the gastrointestinal tract into the bloodstream, thereby precipitating the genesis of immune-mediated pathological conditions. Dysbiosis of the gut microbiota, denoting a perturbation in the equilibrium of the bacterial microflora, emerges as a potent causative determinant in the pathogenesis of chronic diseases, such as metabolic diseases (diabetes mellitus, obesity, cardiovascular diseases), autoimmune diseases, necrotizing enterocolitis, skin diseases, Crohn’s disease and ulcerative colitis [27,46]. It has been discerned that individuals afflicted with Inflammatory Bowel Disease (IBD) manifest more pronounced variations in the composition of their gut microbiota [57]. A significant decrease in beneficial intestinal bacteria (*Bifidobacterium longum, Eubacterium rectale, Faecalibacterium prausnitzii, Roseburia intestinalis*), as well as enrichment in several harmful bacteria, connected to intestinal inflammation and alterations of epithelial cells permeability (e.g., *Escherichia-Shigella, Bacteroides* spp., *Mycobacterium avium paratuberculosis, Clostridium difficile, Helicobacter* spp., *Campylobacter* spp., *Salmonella* spp., *Yersinia* spp. or *Listeria* spp.), has been recognized in patients with ulcerative colitis. Since it has been shown that stimulation of human peripheral blood mononuclear cells with *F. prausnitzii* induces the production of anti-inflammatory IL-10 and inhibits the production of inflammatory cytokines, such as IL-12 and IFN-γ, a decreased amount of this species exposed patients to a higher risk of intra-intestinal inflammatory processes. In UC patients, the recovery of colonization with the *F. prausnitzii* population after relapse was associated with maintenance of clinical remission [48]. *Fusobacterium nucleatum (F. nucleatum)* is commonly found in the oral cavity and intestinal mucosa of humans. A strong association between *F. nucleatum* and intestinal conditions like inflammatory bowel disease (IBD) and colorectal cancer (CRC) has been indicated by researchers [58]. It has been discovered that the bacterium contributes to these diseases by fostering intestinal inflammation and triggering the release of inflammatory substances. In the case of ulcerative colitis (UC), *F. nucleatum* exacerbates the condition by influencing the polarization of M1 macrophages. This bacterium has been demonstrated to worsen UC by promoting damage to intestinal epithelial cells and increasing the secretion of inflammatory cytokines such as IL-1β, IL-6, IL-17F, and TNF-α. What is more, it specifically targets caspase activation and recruitment domain 3 (CARD3) through NOD2, activating the IL-17F/NF-κB pathway. Consequently, *F. nucleatum* coordinates a molecular network involving CARD3 and IL-17F to regulate the progression of ulcerative colitis [59]. In pediatric patients with IBD, there has also been a significant increase in populations of *Veillonella parvula, Streptococcus parasanguinis, Haemophilus parainfluenzae, Granulicatella paradiacens, Ruminococcus flavefaciens,* and *Dorea massiliensis*. Within the identical cohort, a significant reduction in the abundance of *Ruminococcus flavefaciens* and *Alistipes massiliensis* populations has been documented [60]. At the onset of the disease, a higher population of gut *Ruminococcus torques* and *Ruminococcus* as well as higher transcriptional activity correlated with an abundance of *Clostridium hathewayi, Clostridium bolteae,* and *Ruminococcus gnavus* has been noted in UC patients [61]. Furthermore, it has come to attention that there is an augmentation in the population of *Eubacterium rectum* and *Intestinibacter* spp., concomitant with a diminishment in the abundance of *Akkermansia municiphila* within the same context [62]. Patients diagnosed with Ulcerative Colitis (UC) also exhibit notable alterations in their mycobiome composition. Specifically, during disease exacerbation, there is a discernible elevation in the ratio of fungal diversity to bacterial diversity, alongside an augmented prevalence of *Candida albicans* and the yeast *Malassezia restricta*, contrasting with both a healthy control cohort and UC patients in a state of remission [63]. It has been discovered that the glycoprotein cell wall elements found in fungi, namely chitin, β-glucans, and mannans, have the ability to initiate the innate immune response. This activation occurs through various receptors, including dectin-1 (a C-type lectin receptor), TLRs, components of the complement system, and members of the scavenger receptor family (specifically CD5, SCARF1, and CD36). The activation of these receptors sets off a series of immune reactions involving molecules such as CARD9, IL-17, IL-22, ITAM, NFAT, and NF-κB [64] (Figure 2).

## 3. The Molecular Role of the Gut Microbiome in the Pathogenesis of Ulcerative Colitis

Ulcerative colitis is associated with alterations in gut microbiota diversity, leading to changes in gut metabolomic and metagenomic profiles [65]. The gut microbiome plays an essential role in the progression of inflammation. Dysfunction in interactions between gut microbiota and epithelial cells have a significant role in UC pathogenesis. Patients with UC show an increased correlation between gut dysbiosis and the expression of inflammatory genes in epithelial cells, leading to dysregulation in immune response [66,67].

Maintaining gut homeostasis relies on mutual interactions between microbes and host immune cells. While microbiota is influenced by immunological factors such as defensins, IgA, or antibacterial lectin RegIIIγ [68,69,70,71], microbes also have an impact on the host’s mucosal immunity. The gut microbiota contributes to inducing the expansion of regulatory T cells that reduce inflammation, regulating activation of NF-kB, and favoring the differentiation of naive T cells into T helper 17 (Th17) and T helper 1 (Th1) cell subsets. Moreover, it stimulates the anti-inflammatory cytokine IL-10 and reduces pro-inflammatory cytokines. Such processes can be promoted by genera like *Clostridia*, *Bacteroides*, *Bifidobacterium, Lactobacillus,* and *Faecalibacterium* [71,72]. The respective contributions of commensal-driven regulatory T cells exhibit variations depending on the experimental models employed to investigate inflammatory processes. While in murine models exposed to mucosal damage, Th17 cells promote tissue healing, in models with diminished regulatory T (Treg) cell populations, both Th1 and Th17 cells, along with IL-23-dependent innate lymphoid cells, exacerbate colitis. It is plausible that inflammatory bowel diseases in humans may similarly arise due to a commensal-mediated imbalance of lymphoid cell subsets that leads to abnormal inflammatory response [71].

The mucus barrier overlying the epithelium is a crucial element in providing an environment for commensal microbes’ colonization. Abnormalities in its structure play a significant role in inflammation onset. Typically, in active UC, the upper crypt goblet cells are depleted, human IgGFc binding protein (FCGBP), zymogen granule protein 16 (ZG16), and calcium-activated chloride channel regulator 1 (CLCA1) are decreased and MUC2 protein is significantly reduced. This results in the weakening of the colonic mucus barrier, contributing to UC development [73,74].

**Figure 3:**
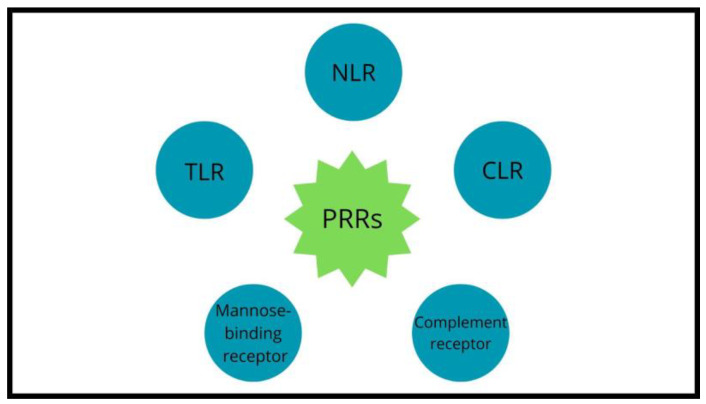
Pattern-recognition receptors’ classes.

The mutual impact between the host’s immune system and microbes is possible due to pattern-recognition receptors (PRRs) such as Toll-like receptors (TLRs), NOD-like receptors (NLRs), mannose-binding receptors, complement receptors and C-type lectin receptors (CLRs) expressed on the intestinal epithelial cells and innate immune cells (Figure 3).

Microbial molecules, called microbe- or pathogen-associated molecular patterns (MAMPs or PAMPs), of both commensal and pathogenic microbes, are recognized by PRRs. These receptors detect bacteria and facilitate the transmission of signals to the host, initiating the recruitment of additional immune cells. That leads to the elimination of the bacteria from the system [75]. PRRs’ responses to MAMPs result in the induction of signaling pathways that initiate a molecular defense against the detected microorganisms. The MAMP-PRR-triggered signaling cascades include the activation of NF-κB–inhibitor of NF-κB kinase (IκBK) and mitogen-activated protein kinase (MAPK) systems, mediated by transient posttranslational protein modifications, transmitting signals from the cell’s surface to its nucleus. The ultimate outcome of these interactions and the ensuing signaling pathways rely on the specific microorganism involved and the responsiveness of the host cell [76]. Activation and translocation of NF-κB mediated by MAMPs/PAMPs promote the expression of inflammatory genes but also genes involved in tissue repair, regeneration, and angiogenesis [77]. Bacteria use several mechanisms to alter PRR function. Strategies such as structural modification of MAMPs, releasing virulence substances, degradation of signaling components, mimicry of adaptors, and epigenetic regulation allow bacteria to manipulate the PRR signaling [78]. Appropriate responses to commensals and pathogens are crucial for maintaining intestinal homeostasis. Abnormal activation of PRRs against commensal bacteria plays a major role in IBD etiology [79,80]. TLRs and CLRs can be activated by dendritic cells (DCs) mediating the destruction of the intestinal barrier. Lamina propria DCs engage with the gut environment to sustain a homeostatic state by producing protective substances, decreasing proinflammatory processes, and inducing the development of adaptive immune tolerance. They induce class switching of B lymphocytes into IgA which is involved in controlling the growth and composition of the enteric microbiota [81]. DCs are one of the significant sources of pro-inflammatory mediators such as cytokines, reactive oxygen species (ROS), and nitrogen intermediates. Increased activation and maturation of dendritic cells contributes to the initiation of inflammation in UC [46,82]. Also, inflammation leads to loss of immunological tolerance maintained by a low expression of CD80 and CD86. In the presence of the microbial environment, immature DCs mature, producing the pro-inflammatory cytokines IL-6, IL-12 and IL-18, and influencing T cell differentiation towards Th1, Th2, and Treg. Intestinal DCs in IBD patients present increased amounts of CD80 and CD40. What is more, recent findings suggest that alterations in the cell surface components of Lactobacilli can influence the immunoregulatory responses of DCs which might pave the way for a targeted therapeutic approach in IBD [83,84]. 

Macrophages also contribute to UC pathogenesis as they mediate microbial defense and are involved in interactions between IBD and the microbiome, though the mechanism is not yet well understood [85]. Studies reveal the role of IL-23 in macrophage bacterial clearance by regulating PRR levels [86]. Macrophages can be divided into two groups according to their functions and phenotypes—M1 and M2 types. Bacterial lipopolysaccharide (LPS) alone or with interferon-γ (IFN-γ), granulocyte–macrophage colony-stimulating factor (GM-CSF), and other cytokines of Th1 lymphocytes induce differentiation into pro-inflammatory M1 macrophages. M1 macrophages promote inflammation through releasing the pro-inflammatory cytokines, NO, and ROS. Conversely, M2 macrophages repair damaged tissue, restore the intestinal barrier, and release anti-inflammatory factors. Studies indicate that dysfunction and imbalance in macrophage polarization can induce inflammatory disorders [85]. Microbes’ effects on macrophage polarization varies. *Fusobacterium* and *Enterococcus faecalis* promote M1 macrophage induction. On the contrary, *Lactobacillus* has a suppressive effect on M1 macrophage activity, *Bacteroides fragilis,* and *Clostridia,* and indirectly *Helicobacter hepaticus* induce M2 polarization [87].

Another important aspect of UC pathogenesis is bacteriophages. The gut virome in UC is altered in comparison to healthy controls [88,89]. The presence of *Caudovirales* phages in rectal mucosa is linked to gut inflammation in UC, as phages are one of the key players in microbiome shaping in IBD. Bacteriophages, including *Escherichia*, *Enterobacteria, Lactobacillus,* and *Bacteroides* phages, are more abundant in UC mucosa than healthy controls, modulating the inflammatory response. They can stimulate IFN-γ through the TLR9 receptor [90,91]. Bacterial lysis caused by bacteriophages can lead to the triggering of an inflammatory response through the activation of PRR by nucleic acids, proteins, and lipids that are released from damaged bacterial cells [92].

The intestinal barrier of patients with UC presents reduced mucus layer and goblet cells compared to the healthy gut, resulting in increased intestinal permeability [93,94]. This condition leads to molecular changes in intestinal epithelial cells. G protein-coupled receptors (GPR) and Toll-like receptors (TLR) are activated by bacterial molecules such as short-chain fatty acids (SCFA). Alteration in the epithelial barrier promotes gut inflammation by increasing stimulation of the IL-17 receptor A (IL-17RA) and accumulating granulocytes in the mucosa [95]. IL-17 is a cytokine produced by T helper (Th17) cells. Subsets of IL-17—IL-17A and IL-17F can trigger the expression of various proinflammatory cytokines and chemokines in different cells thanks to the widespread expression of their corresponding receptor, IL-17RA [96]. Patients with IBD show mucosal secretion of IgG antibodies and mucosal T-cell responses against commensal microbiota [72].

Studies on biopsies obtained from the colons of patients with a diagnosis of UC have revealed that the pro-inflammatory environment in UC gut mucosa is linked to increased gene regulation and expression of cytokines such as IL1A, IL1B, IL4, IL6, IL8, IL17, CSF2, and CSF3, chemokines like CXCL11 and CCL19, secreted factors such as NOS2A, and molecules related to cellular migration- SELE and SELP. Expression of anti-inflammatory factors such as cytokines IL13, CSF1, chemokines CCL3 and CCL5, and molecules SMAD7, BCL2, CYP7AI, AGTR1, and FASLG which are involved in intracellular signaling and apoptosis, is decreased [97].

These immunological processes alter responses to bacterial DNA. However, in UC patients, some probiotic bacteria stimulate anti-inflammatory processes, while pathogenic bacteria stimulate pro-inflammatory responses. This suggests that the ability to distinguish bacterial DNA is not completely lost, enabling the use of probiotics to modulate inflammation among UC patients [97,98].

## 4. The Microbiome as a Moderator in Ulcerative Colitis Development

Epidemiological observations have linked factors that can influence the microbiota such as breastfeeding, hygiene hypothesis, antibiotic use, diet, cigarette smoking, and episodes of infectious gastroenteritis, to the development of IBD [99].

There is some evidence that breastfeeding plays a protective role against ulcerative colitis development. Breast milk is a complex biofluid comprising numerous antimicrobial and immunomodulatory components, prebiotics and probiotics [100]. It contains bacteria species such as *Lactobacillus gasseri*, *Lactobacillus rhamnosus*, *Lactobacillus plantarum*, *Lactobacillus fermentum* and *Bifidobacteria* [101]. Human milk probiotics impact the development of the immune system, promote gut health by strengthening the gastrointestinal mucosa and enhancing the production of protective antibodies (sIgA). *Lactobacillus reuteri* has been found to stimulate type 3 innate lymphoid cells (ILCs) in the small intestinal lamina propria to enhance IgA production [102]. ILC3s are the first line of defense against various pathogens and have the ability to secrete IL-17 and/or IL-22 [103]. There is accumulating evidence that probiotic bacteria generate FoxP3 T-cell responses in the small intestine [104]. They also play a crucial role in the formation of the lymphoid tissue associated with the gut (GALT) [105]. Additionally, probiotics help prevent gastrointestinal infections by countering a pathogenic microbiome through modifying intestinal conditions and competing with pathogens for resources and adhesion sites on intestinal surfaces. *Lactobacilli* were also found to demonstrate increased mucin expression [106]. Furthermore, *Lactobacillus fermentum* enhances IFNγ and Th1 cytokines secretion [107,108], as well as CD56^+^CD8^+^ NK cells’ activation [109]. Breastfeeding significantly reduces the number of gastrointestinal infections in infants [110]. Systematic reviews by Barclay et al. and Klement et al. demonstrate a protective effect of breastfeeding in the development of IBD, but highlight the need of more large, well-designed studies [25,111]. A 2018 meta-analysis proves the protective impact of breastfeeding on ulcerative colitis development [112]. Breastfeeding duration showed a dose-dependent correlation, with the most significant reduction in risk when breastfed for at least 12 months as compared to shorter period.

According to the hygiene hypothesis, people raised in a sanitary environment have increased incidence of immune-related diseases, but its role in IBD pathogenesis is still unclear [113]. Improved hygiene is thought to result in a limited exposure to microorganisms and that exposure is necessary for the development of the immune system and establishing the balance between pro-inflammatory and regulatory cells [114]. The meta-analysis by Cholapranee et al. showed strong protective correlation between IBD and lower environmental hygiene—bedroom sharing, exposure to farm animals and pets, and multiplicity of siblings [115]. The possible mechanisms involve childhood exposure to infectious antigens, which can influence the type of immune response, antigenic competition and the impact on regulatory T-cell function or modifications in the gut microbiome. A number of studies confirmed the role of environmental hygiene in IBD development, but the susceptibility of subjects is influenced by ethnicity [115,116,117].

Antibiotic use prior to diagnosis has been connected to the development of IBD and is thought to be linked to the impact on the gut microbiota and immune regulation in genetically susceptible patients. The study by Shaw et al. showed odds for developing IBD in childhood increased by 2.9 in subjects who have used antibiotics in the first year of life [118]. Another study demonstrated that patients with IBD were more likely to have received antibiotics 2–5 years before their diagnosis [119]. However, in a meta-analysis by Ungaro et al., antibiotics appear to increase the odds of being diagnosed with Crohn’s disease but not ulcerative colitis [120]. The background of this correlation is still unclear, but studies have suggested a difference in the microbiota between CD and UC patients [121]. It is possible that antibiotics are more likely to alter the microbiome in a way that predisposes to CD as opposed to UC or that changes in the microbiota play a more significant role in the development of CD than in UC [120].

Given the substantial impact of food and nutrients on the gut microbiome, there is a growing interest in examining the link between diet and UC development. The composition and diversity of the gut microbiota can be influenced by diet [122]. A diet with high meat intake increases *Bacteroides* spp., *Alistipes* spp., and *Bilophila* spp. and decreases the beneficial bacteria *Lactobacillus* spp., *Roseburia* spp., and *E. rectale* [123,124]. Diets rich in fats reduce the amount of *Bacteroidetes* and increase the abundance of *Firmicutes* [125,126]. A number of studies have shown an altered SCFAs profile in subjects with IBD [127,128]. The SCFAs level is mainly regulated by the gut microbiome, with *Firmicutes* mainly producing butyrate and *Bacteroides* mainly producing acetate and propionate [129]. SCFAs’ mechanisms of action include local, immune, endocrine effects, and alteration of the microbiome–gut–brain axis [130]. The major SCFA signaling pathways are inhibition of histone deacetylases and activation of G-protein-coupled receptors [131]. Fermentation of fiber to SCFAs decreases pH levels, increases fecal acidification, and increases the growth and diversity of the gut microbiome [132]. SCFAs have been shown to alter chemotaxis and phagocytosis, induce reactive oxygen species, change cell proliferation and have anti-inflammatory and antimicrobial effects [131]. A systematic review showed an association between high dietary intake of total fats, PUFAs, omega-6 fatty acids, and meat, with an increased risk of ulcerative colitis development. On the contrary, a high vegetable intake was associated with decreased UC risk [133], which was also confirmed in another study [134]. A high intake of long-chain n-3 PUFAs seemed to be correlated with a reduced risk of UC [135], as well as high caffeine intake [134]. N-3 PUFA supplementation results in a decrease in *Faecalibacterium*, often associated with an increase in the *Bacteroidetes* and butyrate-producing *Lachnospiraceae* [136]. In another study, it resulted in an increased abundance of *Bifidobacterium* and *Oscillospira* genera, associated with a reduction in *Coprococcus* [137]. A study by Balfegó et al. revealed a significant decrease in *Firmicutes* species and the *Firmicutes*/*Bacteroidetes* ratio and an increase in *E. coli* concentrations [138]. A Japanese study suggested that the number of patients with IBD started to increase more than 20 years after an increased daily consumption of meat, fats, and dairy products, and after a decreased consumption of rice [139]. A study by Geerling et al. concluded that high intake of mono- and polyunsaturated fat and vitamin B6 may enhance the risk of developing UC [140]. An association between unhealthy dietary patterns and an increased risk of UC was found in a study by Rashvand et al. [141]. Two meta-analyses found that soft drink consumption and sucrose intake were correlated with increased risk of UC development, and tea consumption with a decreased risk [142,143]. Another meta-analysis indicated that consumption of vegetables and fruit may play a protective role against UC [144]. The meta-analysis of nine studies showed a significantly greater risk of IBD among meat consumers [145]. However, a study by Ananthakrishnan et al. found that diet is associated with risk of CD, but not UC [146]. Dietary patterns or nutrient groups were not associated with ulcerative colitis.

Cigarette smoking has a complex interaction with IBD, currently being widely accepted as a protective factor against UC [147,148,149,150]. This protective effect is temporary, since the risk of UC development increases after smoking cessation, compared with never-smokers [151]. Studies are ambiguous whether it may have a beneficial influence on the course of the disease [21]. According to the study by Lunney et al., current smokers with UC are more likely to have a less severe disease course than nonsmokers [152]. However, a study by Blackwell et al. and meta-analysis by To et al. showed that smokers and non-smokers have similar outcomes regarding flares of disease activity, thiopurine use, development of pouchitis, corticosteroid dependency, hospitalization and colectomy [153,154]. What is more, there is a link between active smoking and extra-intestinal manifestations of UC, such as skin disorders or joint manifestations [155]. The underlying protective mechanism potentially includes changing the humoral and cellular immunity, cytokine levels, gut motility, and oxygen free radicals [156]. It has been shown that cigarette smokers have a lower amount of *Bifidobacterium* bacteria compared with non-smokers, and it increases after smoking cessation [157].

Infectious gastroenteritis is a recognized factor that can exacerbate the clinical course of IBD. In a study by Porter et al. the episode of infectious gastroenteritis has been shown to increase the risk for the development of IBD by 40% [158]. Another study showed a two- to four-fold increased risk of IBD after an episode of gastroenteritis [159].

**Figure 4:**
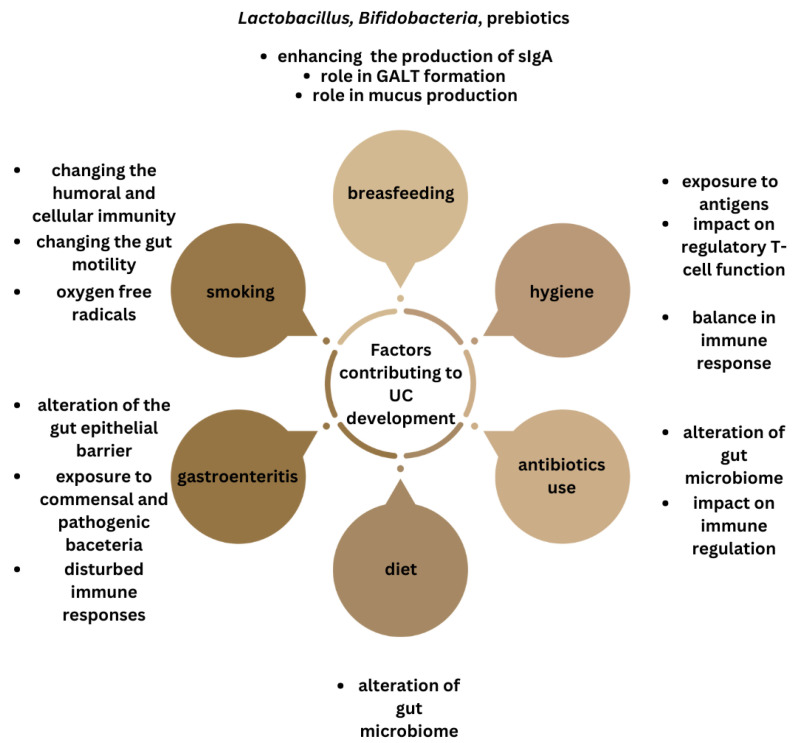
Summary of the factors influencing gut microbiota, linked with ulcerative colitis development.

In summary, there is evidence suggesting that environmental factors can impact the gut microbiota and trigger immune responses in individuals genetically predisposed to IBD. While research has explored these effects in animal models, human studies are limited. Future studies in humans, particularly those with known IBD genetic risk factors, will provide a clearer understanding of these relationships (Figure 4).

## 5. Treatment

The current treatment for UC focuses on achieving and sustaining symptom remission, minimizing complications, and improving patients’ quality of life [38]. The primary drug classes employed include aminosalicylic acid agents, glucocorticoids, and immunosuppressive agents [4,14]. Aminosalicylic acid agents can help control UC symptoms but may lead to gastrointestinal side effects [160]. Glucocorticoids are effective in inducing remission but come with potential side effects such as osteoporosis and muscle weakness [41,42]. Immunosuppressants are reserved for cases where other treatments fail but can result in serious adverse effects if used long-term [43]. Notably, recent advancements in the development of monoclonal antibodies and recombinant proteins targeting cytokines represent a significant breakthrough in UC treatment [44]. Targeted drugs for UC include anti-TNF-α monoclonal antibodies, integrin antagonists, IL-12/IL-23 antagonists, JAK inhibitors, and SIP receptor agonists. The American Gastroenterology Association (AGA) recommends using infliximab, adalimumab, golimumab, vedolizumab, tofacitinib or ustekinumab to induce or maintain remission in adult patients with moderate or severe UC [45]

Modulating the gut microbiome via dietary components represents a promising avenue for enhancing therapeutic interventions in UC. Ester compounds, formed by combining fructooligosaccharides (FOS) with short-chain fatty acids (SCFAs), were examined in terms of their impact on gut microbiota in UC patients [161]. Butyrylated fructooligosaccharides (B-FOS) and propionylated fructooligosaccharides (P-FOS), in particular, significantly promoted *Bifidobacterium* growth while inhibiting *Clostridium* and *Klebsiella*. This research underscores the potential therapeutic utility of B-FOS and P-FOS in mitigating UC-related inflammation and restoring the gut microbiome.

A review on the efficacy of probiotics for the induction of remission in active UC suggested that incorporating a probiotic into standard therapy can enhance overall remission rates in UC patients [162]. The effect of synbiotic therapy on the disease activity in UC patients was evaluated and statistically significant improvement was observed in the clinical and endoscopic activity levels after 8 weeks of treatment [163], which is in line with further studies [164].

The clinical efficacy of fecal microbiota transplantation (FMT) in UC patients was examined in a prospective study [165]. FMT led to a significant reduction in UC patients’ symptoms, including diarrhea and abdominal pain. Male patients showed dominant *Clostridiales* and *Desulfovibrionaceae* in their gut microbiota, which decreased following FMT. In contrast, the abundance of *Prevotella*, *Lactobacillus*, and *Bifidobacterium* increased in the male group. Female patients had higher levels of *Escherichia-Shigella*, *Desulfovibrionaceae*, and *Staphylococcaceae* in their gut microbiota before FMT, which decreased after the procedure. The abundance of *Porphyromonadaceae*, *Prevotella*, *Lactobacillus*, and *Bifidobacterium* increased in the female group. These findings suggest that FMT improved UC symptoms in both male and female patients, and these improvements may be linked to changes in their gut microbiota, which corresponds with other studies [27,166]. A recent meta-analysis also showed FMT for treating patients with active UC as a promising therapy, with a high rate of clinical remission [167]. Results of an open-labelled randomized controlled trial showed that a combination of FMT and anti-inflammatory diet effectively induced UC remission and further sustained with an anti-inflammatory diet [168].

The influence of FMT on the immune response in individuals with UC and the potential underlying mechanisms are not currently well understood. One study aimed to evaluate alterations in serum cytokine levels and their association with disease activity following FMT in patients with active UC [169]. Sixteen individuals with active UC underwent three FMT sessions from a single donor, with significant reductions in IL-1Ra, IL-6, IP-10, ENA-78, MEC, VCAM-1, and G-CSF observed after the second FMT. This finding is in line with previous research [170]. IL-6, IL-1Ra, IP-10, VCAM-1, and G-CSF exhibited positive correlations with inflammatory markers, suggesting that FMT may influence the host immune response. The study suggests a biomarker potential of IL-6, IL-1Ra, IP-10, VCAM-1, and G-CSF for evaluating the effectiveness of FMT in treating UC. On the other hand, a study on short-term cytokine changes in UC patients undergoing FMT revealed no significant differences in cytokines IL-2, IL-4, IL-6, IL-10, IL-17A, IFN-γ, TNF, TNFR-1, TNFR-2, MCP-1 nor G-CSF levels, at three days post-FMT, irrespective of response or non-response groups [171]. The inconsistency in research findings underscores the need for further investigations in this field.

**Figure 5:**
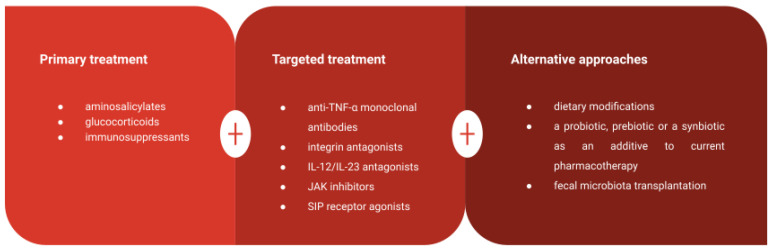
Summary of various available treatment approaches in UC patients.

The diversity of drug classes for UC management highlights the need for identifying biomarkers to predict treatment response. Existing evidence on the utility of combining biologics and immunomodulators in UC patients, particularly with newer agents, and the optimal treatment targets for UC, remains limited (Figure 5).

## 6. Conclusions

The meticulous maintenance of a healthy and equitably balanced microbiota represents an elemental imperative in the preservation of an optimal gut milieu. A suitable intestinal microbiome composition, characterized by an elevated presence of advantageous anaerobic microorganisms juxtaposed with a residual contingent of pathogenic and intestinal non-dominant bacteria, endows the organism with the capacity to fine-tune immune responses, govern the integrity of the intestinal mucosal barrier, and serve as a bulwark against the onslaught of infectious agents and the inception of systemic diseases, including ulcerative colitis.

Observational studies have associated factors like breastfeeding, hygiene, antibiotic use, diet, smoking, and infectious gastroenteritis with the development of IBD. There is evidence indicating that these factors can affect the gut microbiota and alter immune responses in individuals with a genetic predisposition to ulcerative colitis, but future research is needed to provide a clearer understanding. 

Current therapeutic approaches primarily focus on achieving symptom remission and minimizing complications. Notably, recent advancements in monoclonal antibody therapy have opened promising avenues for managing UC. Additionally, the modulation of the gut microbiome through dietary components, probiotics, and FMT shows considerable potential for enhancing UC treatment strategies, particularly in cases where first-line therapies prove ineffective. Nonetheless, there is an ongoing need for the identification of biomarkers to predict treatment response and the optimization of treatment strategies, both of which remain critical areas of research in UC management.
